# Estimated Changes in Manufacturer and Health Care Organization Revenue Following List Price Reductions for Hepatitis C Treatments

**DOI:** 10.1001/jamanetworkopen.2019.6541

**Published:** 2019-07-05

**Authors:** Sean Dickson, Ian Reynolds

**Affiliations:** 1 Pew Charitable Trusts, Washington, DC; 2 now with West Health Policy Center, Washington, DC

## Abstract

**Importance:**

Pharmaceutical manufacturers rarely reduce drug list prices, but 3 expensive treatments for hepatitis C experienced significant list price reductions in 2018. Understanding the impetus for these price reductions could inform policies to reduce drug spending.

**Objective:**

To estimate the differences in manufacturer and health care organization revenue from the Medicare Part D program following list price reductions for hepatitis C treatments, accounting for manufacturer discounts to eligible health care organizations under the 340B drug discount program and manufacturer rebates to pharmacy benefit managers.

**Design, Setting, and Participants:**

A cross-sectional analysis of Medicare Part D claims for hepatitis C treatments in 2016 was conducted. Data analysis was performed in February 2019. Using the observed price changes from 2018, manufacturer and 340B health care facility net revenues for each drug were estimated before and after the price change based on 2016 use, adjusting for estimated 340B health care organization discounts and pharmacy benefit manager rebates. The 340B health care organizations include hospitals, clinics, and other organizations that meet federal standards to participate in the 340B program and were actively enrolled in the 340B program from January 1 to December 31, 2016. Manufacturer discounts to 340B health care organizations are based on the price of a drug before rebates to pharmacy benefit managers, and a reduced list price would reduce discounts to 340B health care organizations. Health care organization–level claims data were obtained from the Medicare Part D Provider Utilization File, and health care organizations were matched to Health Resources and Services Administration Office of Pharmacy Affairs Information System to identify 340B-eligible health care organizations. Eligible claims included claims for ledipasvir with sofosbuvir (Harvoni; Gilead Sciences Inc), sofosbuvir with velpatasvir (Epclusa; Gilead Sciences Inc), and elbasvir with grazoprevir (Zepatier; Merck). Health care organizations were considered 340B eligible if their practice address was a registered 340B entity for the entirety of 2016.

**Main Outcomes and Measures:**

Discounts to 340B health care organizations and pharmacy benefit managers for each drug before and after the price change were the primary outcomes. Other outcomes included per-treatment and aggregate manufacturer and 340B health care organization net revenues for each drug before and after the price change and the share of claims prescribed by 340B health care organizations for each drug. Per-treatment manufacturer net revenues were estimated for 340B health care organizations, non-340B health care organizations, and a weighted average revenue across health care organization types.

**Results:**

The 3 hepatitis C treatments evaluated had 30% to 41% of claims prescribed by 340B-eligible health care organizations, greater than the 14% 340B prescribing rate for all Medicare Part D drugs. Based on use data from 2016, list price reductions for hepatitis C treatments in 2018 were estimated to have increased aggregate manufacturer net revenues for 3 treatments by $181.9 million—a 28% increase. Aggregate 340B health care organization net revenues were estimated to have been $181.9 million lower—a 74% decrease.

**Conclusions and Relevance:**

List price reductions for hepatitis C treatments may have increased drug manufacturer net revenues, owing in part to lower discounts provided under the 340B program and the high share of sales subject to those discounts. Policymakers should consider the role of 340B discounts when evaluating policies to reduce drug spending.

## Key Points

**Question:**

Did drug manufacturer net revenues increase after list price reductions for hepatitis C treatments, accounting for changes in pharmacy benefit manager and 340B drug pricing program discounts?

**Findings:**

In this cross-sectional analysis of 112 630 Medicare Part D hepatitis C treatment claims for 3 drugs in calendar year 2016, estimated manufacturer net revenues increased by 28% after manufacturers reduced the list price of these treatments. Net revenues for 340B health care organizations for these 3 treatments were estimated to have decreased by 74% after these list price reductions.

**Meaning:**

Manufacturer drug pricing decisions may be associated with the proportion of sales subject to both pharmacy benefit manager and 340B drug pricing program discounts.

## Teaser

This cross-sectional study examines changes in revenues of manufacturers of drugs for treatment of hepatitis C and health care organizations after list price reductions.

## Introduction

Three hepatitis C (HCV) direct-acting antiviral curative treatments (ledipasvir with sofosbuvir [Harvoni; Gilead Sciences Inc], sofosbuvir with velpatasvir [Epclusa; Gilead Sciences Inc], and elbasvir with grazoprevir [Zepatier; Merck]) experienced price reductions of 60% or more in the second half of 2018.^1,2^ Price decreases for brand-name drugs are rare; even after generic competition, manufacturers of brand-name drugs increase the prices.^3^ Although pharmacy benefit manager (PBM) rebates may offset increases, some argue that such rebates drive price increases.^4^ Concurrently, manufacturers argue that the 340B drug pricing program (340B program), which allows federally designated health care organizations to purchase discounted drugs, drives price increases by reducing manufacturer revenues.^5^ We conversely hypothesize that the combination of PBM rebates and 340B discounts has driven price reductions in the HCV treatment market, as manufacturer revenues are actually greater following price reductions.

The 340B program was established in 1992 following changes in the drug market after the establishment of the Medicaid Drug Rebate Program.^6^ The Medicaid Drug Rebate Program uses a formula to determine the net price of drugs for the Medicaid program, and that formula requires drug manufacturers to extend the best price offered to any commercial purchaser to the Medicaid program.^7^ Following the introduction of the Medicaid Drug Rebate Program, manufacturers were less willing to provide discounts to safety-net institutions and government purchasers because a single discount to one health care organization could reduce the manufacturer’s net revenue for all Medicaid purchases.^8^ To fix this unintended consequence, in 1992, Congress passed a series of exclusions from the best price requirement, identifying a variety of health care organizations and government agencies to which manufacturers could extend discounted pricing without triggering best price. As part of this carve-out, Congress required manufacturers to extend net Medicaid pricing (340B discount) to these identified 340B health care organizations (separate discounts were established for government purchasers). The 340B health care organizations include hospitals, clinics, and other facilities that meet federal standards to participate in the 340B program. Currently, these discounts are at least 23.1% of a brand-name drug’s average manufacturer price,^9^ which approximates the list price for brand-name drugs^10^; these discounts are greater if the manufacturer has increased prices higher than the rate of inflation.^11^

The 340B statute identifies which health care organizations are eligible to participate in the program; the largest share of discounted 340B sales is to disproportionate share hospitals,^12^ a federal hospital status based on the number of Medicaid patients served by the hospital.^13^ Other 340B-eligible health care organizations include federally qualified health centers; health care organizations participating in the Ryan White program, which funds HIV care; family planning health care organizations; and hemophilia treatment centers, among others.^14^

When a 340B-eligible health care organization purchases a drug under the 340B program, the manufacturer must sell that drug to the facility at the discounted price.^15^ When the health care organization bills an insurer for that drug, however, it is not required to bill the insurer at the discounted cost of the drug, allowing 340B health care organizations to bill insurers at the undiscounted rate. The difference between the discounted purchase price and the reimbursed price is retained by the 340B health care organization and is intended to allow those facilities to “stretch scarce Federal resources as far as possible, reaching more eligible patients and providing more comprehensive services.”^16^

Pharmacy benefit managers are contracted entities that administer the prescription drug benefit of health insurance.^17^ Among other functions, PBMs generally are responsible for creating the drug formulary for a given insurance plan, establishing coverage, patient cost-sharing, and use management. In this role, PBMs negotiate with manufacturers for rebates to offset drug costs, using the leverage of formulary inclusion, patient costs, and management of use. Under this model, the PBM will reimburse a pharmacy for the full cost of a drug but receive a rebate from the manufacturer to reduce the PBM’s net cost for the agent. In competitive drug classes, PBM rebates can exceed 50% of the cost of the drug.^18^

When a 340B health care organization is reimbursed by a PBM for the undiscounted cost of a drug, the manufacturer must generally pay the PBM the standard negotiated rebate for that drug to offset the PBM’s costs, even though the manufacturer sold the drug to the 340B health care organization at a discounted cost. If PBM rebates are high and 340B sales are a significant share of total sales, these combined discounts could significantly reduce net manufacturer revenue. In the HCV market, where both of these conditions appear to be true, we hypothesize that the manufacturer may prefer to reduce the list price of the drug, reducing both the 23.1% 340B discount and the PBM rebate payment. The Figure shows a simplified example of these transactions, using a hypothetical $100 drug with a $50 PBM rebate.

**Figure.**
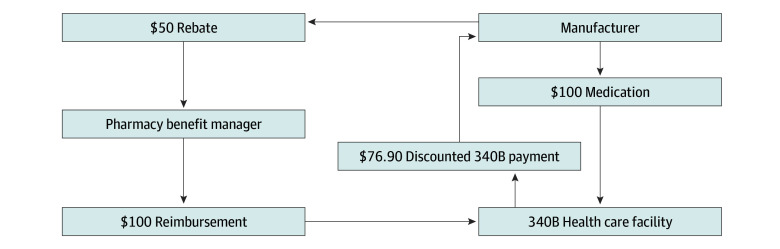
Payment Flow for a $100 Medication Prescribed at a 340B Health Care Organization The discounted payments made by a 340B health care organization to a manufacturer for a hypothetical $100 drug and the rebates paid by the manufacturer to a pharmacy benefit manager when the pharmacy benefit manager reimburses the 340B health care organization for the drug.

## Methods

This analysis determines the share of Medicare Part D HCV treatments that were prescribed at 340B health care organizations in 2016 and uses these market data to estimate manufacturer and health care organization revenues before and after price reductions for HCV drugs. In this section, we first outline our methods to determine the share of prescriptions from 340B health care organizations and then detail our methods to estimate manufacturer and health care organization revenues, both at a per-treatment level and at the aggregate level based on Medicare Part D use. For comparison, we calculate the share of prescriptions from 340B health care organizations for other Medicare Part D drugs, aggregated by therapeutic class. This study was approved by the Pew Charitable Trusts. This study was not submitted for institutional review board approval because it did not involve health care records and all data are publicly available, per the decision guidance from the US Department of Health and Human Services. This study followed the Strengthening the Reporting of Observational Studies in Epidemiology (STROBE) reporting guideline for cross-sectional studies.

### Study Design and Data Sources

We performed a cross-sectional analysis of data from January 1 to December 31, 2016, to model how changes in the cost of HCV treatments would be associated with manufacturer and health care organization revenues during that period. The Medicare Provider Utilization and Payment Data: Part D Prescriber PUF NPI Drug CY 2016 data set was used to identify prescriber-level claims for individual drugs.^19^ This data set is organized by national provider identifier (NPI), which uniquely identifies each prescriber eligible for reimbursement in the Medicare program. Each NPI is associated with drug-level claims information for all drugs prescribed by an individual prescriber; drugs are identified by brand and generic name. Each NPI drug entry includes the number of beneficiaries for whom the drug was prescribed by that prescriber, the number of claims, the number of 30-day fills, the total number of days prescribed, and the total drug invoice cost, among other data. For privacy concerns, only prescribers with 11 or more claims for a particular drug are included in the data set; the number of beneficiaries receiving the prescription is omitted if fewer than 11 beneficiaries received the prescription from a given prescriber.

The Medicare Provider Utilization and Payment Data: Part D Prescriber Summary Table CY 2016 data set was used to match each NPI to full address information.^20^ The Health Resources and Services Administration Office of Pharmacy Affairs Information System (340B-covered entity database) was used to identify facilities that were actively enrolled in the 340B program from January 1 to December 31, 2016.^21^ Only facilities that were actively enrolled for the entire study period were considered to be eligible 340B facilities. This database was also used to determine the 340B-covered entity type associated registered to each address.

The United States Pharmacopeia Medicare Model Guidelines, version 6.0, With Example Medicare Part D Drugs data set was used to identify the therapeutic class for brand drugs.^22^ This version of the data set applied to Medicare Part D benefit years 2015-2017.

### Data-Matching Approach

Addresses by NPI from the Medicare prescriber summary data set were matched to the 340B-covered entity database using address number, street name, city, and zip code; 340B-eligibility status and covered entity type were then matched to the claims data by NPI. This process is similar to the NPI-based matching used to exclude 340B claims from Medicaid rebate reporting^23^ and the address-based matching used to estimate 340B market share in the Medicare Part B program.^24^ Therapeutic class was matched to claims data for brand-name drugs only, using the first word in the brand name; generic drugs were not considered in the therapeutic class analysis.

### Time Period

This cross-sectional study is limited to calendar year 2016. While manufacturer reductions in list prices for HCV treatments occurred in 2018, this study only models the changes in revenue that manufacturers would have realized in 2016 had those price changes been implemented on January 1, 2016. Claims data necessary to model use following the 2018 price changes are not publicly available, and 2016 is the most recent year for which Medicare claims-level data are available.

### Comparators

We compared the estimated manufacturer revenue, 340B health care organization revenue, and combined Medicare and beneficiary drug costs before and after the price reduction for each drug. We also compared the share of brand-name prescriptions prescribed at 340B-eligible health care organizations (340B market share) for the 3 HCV treatments of interest and for each therapeutic class.

### Revenue and Costs Estimation Model

We estimated manufacturer revenue per course of treatment net of rebates for both 340B and non-340B sales before and after the price reduction. We assumed that, before the price reduction, the manufacturer offered rebates to PBMs that achieved a net price equal to the new list price following the price reduction. This assumption is consistent with one manufacturer’s assertion that the reductions “more closely reflect the discounts that health insurers and government payers receive today”^1^ and another manufacturer’s statement that the reduction was based, in part, on “the gap between list price and actual discounted (net) prices paid in the market.”^2^ This assumption can also be understood as modeling revenues and costs if the manufacturer had chosen to achieve the price reduction through a PBM rebate rather than a list price reduction. We assumed that after the list price reduction, the manufacturer offered no further rebates to PBMs. The 340B discount is 23.1% of a drug’s average manufacturer price^9^; we used each drug’s list price as a proxy for the average manufacturer price.^10^ We present the net manufacturer revenue per course of treatment for each drug for both 340B and non-340B sales as well as the weighted average net revenue per course of treatment for each drug adjusted for its share of 340B and non-340B sales, as calculated from the Medicare Part D 2016 use data.

To estimate 340B health care organization revenue per treatment, we assumed that 340B health care organizations are reimbursed by insurers at the list price for the drug. Revenue is the difference between the list price and the 340B price, or 23.1% of the list price.

Total combined Medicare and beneficiary costs per treatment were estimated as the list price of the drug net of PBM rebates. We did not separately calculate costs for Medicare and beneficiaries because beneficiary costs will vary significantly based on plan design and other medications used by the beneficiary.

Aggregate revenues and costs for each drug were calculated by multiplying the number of treatments prescribed in 2016 at 340B and at non-340B facilities by the relevant per-treatment price. Because the standard treatment course for each of the 3 drugs considered is 12 weeks and the total number of claims for HCV treatment in the data (112 630.0) varied by less than 1% from the number of 30-day fills for HCV treatment (112 696.6), we assume that a course of treatment equals 3 claims. Although a limited number of patients may receive treatment courses of shorter or longer duration, these variations in treatment duration are included in the total number of claims assessed, and simplifying the division of claims into standard courses of treatment does not affect the aggregate revenues and costs presented. We present findings by course of treatment rather than claims because the costs of HCV drugs are generally discussed in terms of the total cost of treatment.

### 340B Prescription Volume and Market Share

We calculated the share of prescriptions prescribed at 340B facilities for each of the 3 HCV treatments as well as for all Medicare Part D prescriptions (brand name and generic) and for each therapeutic class (brand name only). For the 3 HCV treatments considered herein, we calculated the share of 340B-eligible claims prescribed at each type of 340B-covered entity facility. For ease of interpretation, we combined several of the covered entity types into categories: (1) consolidated health centers and federally qualified health center (FQHC) look-alikes; (2) all 4 types of Ryan White entities (Ryan White); (3) sole community hospitals, critical access hospitals, freestanding cancer hospitals, and rural referral centers (other hospitals); and (4) black lung programs, Native Hawaiian health care programs, children’s hospitals, tuberculosis programs, and urban Indian health centers (other).

### Data Analysis

Analysis was performed using Stata SE, version 14.2 (StataCorp). Data analysis was performed in February 2019.

## Results

### Distribution of 340B-Eligible Prescribers

Of the 1.3 billion drug claims assessed, 14.0% of the agents were prescribed at a 340B-eligible facility. Among the 2913 unique drugs assessed (brand name and generic), 1297 brand-name drugs (45.5%) were matched to a therapeutic class. Across therapeutic classes, the share of 340B-eligible prescriptions ranged from 6.3% (ophthalmic agents) to 40.8% (antivirals). Table 1 displays the share of 340B-eligible prescriptions for therapeutic classes with more than 500 000 total claims for all brand-name drugs within the class in 2016.

**Table 1.** Share of Medicare Part D Brand-Name Drugs Prescribed at 340B-Eligible Health Care Organizations by Therapeutic Class in 2016

Therapeutic Class^a^^,^^b^	340B-Eligible Share, No./Total No. (%)^c^
Ophthalmic agents	6/97 (6.3)
Hormonal agents, stimulant/replacement/modifying (thyroid)	5/51 (9.9)
Antidementia agents	4/36 (10.1)
Antibacterial agents	1/11 (10.1)
Antidepressant agents	2/15 (11.5)
Antigout agents	1/7 (12.1)
Therapeutic nutrients/minerals/electrolytes	4/36 (12.1)
Analgesic agents	4/36 (12.5)
Genitourinary agents	6/46 (12.8)
Cardiovascular agents	23/182 (12.8)
Antipsychotic agents	2/16 (13.1)
Gastrointestinal agents	9/65 (13.5)
Hormonal agents, suppressant (parathyroid)	1/9 (14.1)
Antiaddiction/substance abuse treatment agents	1/9 (14.7)
Hormonal agents, stimulant/replacement/modifying (sex hormones/modifiers)	3/17 (14.9)
Blood glucose regulators	43/277 (15.5)
Blood products/modifiers/volume expanders	17/108 (15.9)
Anticonvulsant agents	9/57 (15.9)
Central nervous system agents	2/15 (16.5)
Immunologic agents	3/18 (16.9)
Respiratory tract/pulmonary agents	46/269 (17.0)
Antineoplastic agents	2/7 (29.0)
Antiviral agents	9/23 (40.8)
Overall^d^	1875/13 429 (14.0)

^a^ United States Pharmacopeia Medicare Model Guidelines, version 6.0, With Example Medicare Part D Drugs.^22^

^b^ Limited to classes with more than 500 000 total prescription claims for all brand-name drugs in the class.

^c^ Claims are expressed in rounded increments of 100 000.

^d^ Includes claims for both brand-name and generic drugs.

For the 3 HCV treatments that reduced list prices in 2018, the 340B-eligible share ranged from 30% (Harvoni) to 41% (Zepatier) in 2016 (Table 2). Of the 340B-eligible claims, 75.1% were prescribed at disproportionate share hospitals, with smaller shares prescribed at FQHCs and other entities.

**Table 2.** Share of Medicare Part D Selected Hepatitis C Virus Treatment Prescriptions Prescribed by 340B-Eligible Health Care Organizations by 340B Entity Type in 2016

Drug^a^	Claims, No. (%)								
	Total	340B	DSH	FQHC^b^	HM^c^	RW^d^	FP^e^	Other Hospital^f^	Other^g^
Epclusa	4360	1586 (36.4)	1296 (29.7)	189 (4.3)	70 (1.6)	0	0	31 (0.7)	0
Harvoni	104 705	31 686 (30.3)	23 625 (22.6)	4414 (4.2)	681 (0.7)	1695 (1.6)	413 (0.4)	640 (0.6)	218 (0.2)
Zepatier	3565	1467 (41.2)	1184 (33.2)	173 (4.9)	54 (1.5)	23 (0.6)	33	0	0

Abbreviations: DSH, disproportionate share hospital; FP, family planning; FQHC, federally qualified health center; HM, hemophilia; RW, Ryan White.

^a^ Generic names of the drugs are velpatasvir (Epclusa; Gilead Sciences Inc), ledipasvir with sofosbuvir (Harvoni; Gilead Sciences Inc), and elbasvir with grazoprevir (Zepatier; Merck).

^b^ Includes community health centers, school-based programs, health care for the homeless programs, migrant health programs, public housing primary care programs, and federally qualified health center look-alikes.

^c^ Comprehensive hemophilia treatment centers.

^d^ Ryan White HIV/AIDS Program grantees.

^e^ Title X grantees.

^f^ Includes critical access hospitals, free-standing cancer hospitals, rural referral centers, and sole community hospitals.

^g^ Includes black lung health care organizations, Native Hawaiian health care programs, children’s hospitals, tuberculosis health care organizations, and urban Indian health centers.

### Per-Treatment Revenues and Costs

Table 3 reports the drug-level per-treatment revenues and costs for each entity before and after the price change. Following the price change, 340B-covered entity revenues for each course of treatment decreased by 60% to 75%; manufacturer revenues from sales to 340B-covered entities increased by 82% to 750% for each course of treatment; net costs to Medicare and beneficiaries remained constant. Following the price change, the average per-treatment manufacturer revenue (weighted by the share of 340B and non-340B sales) increased by 19% to 28% for each course of treatment.

**Table 3.** Changes in Hepatitis C Virus Treatment Prices, 340B Discounts, and Net Revenues and Costs

Drug^a^	List Price	340B Discount^b^	PBM Rebate^c^	Manufacturer per-Patient Revenue			340B Entity per-Patient Revenue	Medicare and Beneficiary Net Cost
				340B	Non-340B	Mean^d^		
**Before Price Reduction, $**								
Epclusa	74 760	17 270	50 760	6730	24 000	17 718	17 270	24 000
Harvoni	94 500	21 830	70 500	2171	24 000	17 394	21 830	24 000
Zepatier	54 600	12 613	32 760	9227	21 840	16 650	12 613	21 840
**After Price Reduction, $**								
Epclusa	24 000	5544	0	18 456	24 000	21 983	5544	24 000
Harvoni	24 000	5544	0	18 456	24 000	22 322	5544	24 000
Zepatier	21 840	5045	0	16 795	21 840	19 764	5045	21 840
**Difference Following Price Reduction, %**								
Epclusa	−68	−68	−100	174	0	24	−68	0
Harvoni	−75	−75	−100	750	0	28	−75	0
Zepatier	−60	−60	−100	82	0	19	−60	0

Abbreviations: HCV, hepatitis C virus; PBM, pharmacy benefit manager.

^a^ Generic names of the drugs are velpatasvir (Epclusa; Gilead Sciences Inc), ledipasvir with sofosbuvir (Harvoni; Gilead Sciences Inc), and elbasvir with grazoprevir (Zepatier; Merck).

^b^ 23.1% of list price.

^c^ Difference between list price prior to price change and list price after price change.

^d^ Weighted mean based on share of Part D prescriptions prescribed at 340B-eligible health care organizations.

### Aggregate Revenues and Costs

Based on use data from 2016, aggregate manufacturer revenues from Medicare Part D for both 340B and non-340B sales of the 3 drugs following the price change were estimated to have been $181.9 million greater—a 28% increase over revenue under the earlier pricing structure (Table 4). The 340B health care organizations were estimated to have had a $181.9 million revenue reduction for these 3 drugs—a 74% reduction from the earlier pricing structure. Combined net Medicare and beneficiary spending would remain constant.

**Table 4.** Changes in Aggregate Net Revenues and Costs Following Price Reductions for HCV Treatment

Drug^a^	Manufacturer Aggregate Revenue^b^	340B Entity Aggregate Revenue	Medicare and Beneficiary Net Cost
**Before Price Reduction, $**			
Epclusa	25 749 927	9 130 073	34 880 000
Harvoni	607 076 821	230 563 179	837 640 000
Zepatier	19 785 443	6 167 757	25 953 200
Total	652 612 191	245 861 009	898 473 200
**After Price Reduction, $**			
Epclusa	31 949 072	2 930 928	34 880 000
Harvoni	779 084 272	58 555 728	837 640 000
Zepatier	23 486 195	2 467 005	25 953 200
Total	834 519 539	63 953 661	898 473 200
**Difference Following Price Reduction $, No. (%)**			
Epclusa	6 199 145 (24)	−6 199 145 (−68)	0
Harvoni	172 007 451 (28)	−172 007 451 (−75)	0
Zepatier	3 700 752 (19)	−3 700 752 (−60)	0
Total	181 907 348 (28)	−181 907 348 (−74)	0

Abbreviation: HCV, hepatitis C virus.

^a^ Generic names of the drugs are velpatasvir (Epclusa; Gilead Sciences Inc), ledipasvir with sofosbuvir (Harvoni; Gilead Sciences Inc), and elbasvir with grazoprevir (Zepatier; Merck).

^b^ Based on weighted average per-treatment revenue by share of Part D prescriptions prescribed by 340B-eligible health care organizations.

## Discussion

Recent manufacturer decisions to reduce the list price of HCV treatments appear inconsistent with a preference to discount high-priced drugs through rebates to PBMs. The concomitant price reductions by multiple manufacturers may reflect factors unique to the HCV market. Manufacturers would not be expected to make pricing decisions that reduce net revenues, implying that list prices reductions were likely financially advantageous to manufacturers. Our analysis suggests that, given the high share of 340B sales within the HCV market, reducing a drug’s list price generates greater net manufacturer revenues than the same price reduction via a rebate mechanism. This increase in manufacturer revenues is because with a high list price, the manufacturer would have to pay both a PBM rebate and a higher 340B discount, but with a lower list price and no rebate, the manufacturer faces only a lower 340B discount.

Analyses have suggested that manufacturers and payers (including PBMs) typically prefer to achieve discounts via rebates rather than list price reductions.^4,25^ Our analysis suggests that this preference may vary based on the share of sales subject to 340B discounts. Because the manufacturer must pay both the 340B discount and a PBM rebate for 340B-eligible prescriptions, manufacturer net revenue is dependent on the market share subject to these stacked discounts. The prevailing preference for rebates over list price reductions may hold at the average 14% 340B market share, but not when the 340B market share exceeds 30% or 40%, as in the HCV market.

This sensitivity to 340B market share is supported by pricing trends within the HCV market before 2018. The launch of initial HCV treatments was characterized by price matching and increases, with the first product, Sovaldi (sofosbuvir; Gilead Sciences Inc), launched at $84 000; the second, Harvoni, launching at $94 500; and the third, Viekira Pak (ombitasvir, paritaprevir, ritonavir, and dasabuvir combination; AbbVie), launching at $83 319.^26^ Subsequent product launches, however, had lower prices even though they targeted more genotypes or had shorter treatment durations than the existing therapies. Epclusa was launched at $74 760,^27^ followed by Zepatier at $54 600^28^ and subsequently Mavyret (glecaprevir with pibrentasvir; AbbVie), with an 8-week treatment duration, at $26 400.^29^ Manufacturers chose to launch later products at lower prices rather than achieve the same net price through a PBM rebate, contrary to the standard theory for manufacturer pricing strategy. Although political attention to HCV pricing may be associated with manufacturers’ pricing strategy, the magnitude of 340B discounts within this class offers a market-based, rather than political, explanation for the decrease in HCV list prices.

While our findings that reduced list prices and 340B discounts increased revenue more than PBM rebates would seemingly apply to all drugs regardless of 340B market share, manufacturers may be more willing to accept lower revenue from 340B sales in exchange for long-term increased revenue from ever-higher list prices. Manufacturers may face pressure from PBMs to increase both prices and rebates, although the reductions in HCV list prices suggest that manufacturers can resist this pressure. Price increases on existing products may also be part of a pricing strategy for future products, establishing price floors near which newly launched products are priced. Investigators from the US Senate Committee on Finance identified this as part of the initial pricing strategy for Sovaldi and Harvoni, stating that “one of Gilead’s considerations for Wave 1 prices, ie, Sovaldi, was the potential to achieve a high price for Wave 2, ie, Harvoni”^26^ and citing company documents that argue “[a]t any price, access for Wave 2 improves as the price for Wave 1 is increased, suggesting that Wave 1 will set a price benchmark against which Wave 2 will ultimately be evaluated.”^26^

The nature of the HCV treatment market offers little strategic benefit to offset price increases with PBM rebates in an attempt to establish a price floor for future product launches. The curative nature of HCV treatments and relatively short treatment duration (8-12 weeks) limits future sales potential, as the number of patients requiring HCV treatment will likely decrease and per-patient sales are limited to a single course of treatment. This regimen differs significantly from the markets for chronic diseases, which may require maintenance therapy for the patient’s life span, or age-related disease markets, where the patient population is expected to grow. In these markets, the strategic value of price increases on existing drugs to serve as price floors for new therapies may exceed the revenue effect of the combined growth of 340B discounts and PBM rebates, even for higher 340B-share markets.

Our analysis suggests that association between the 340B program and manufacturer drug pricing decisions should be reconsidered. Although some manufacturers and other commentators have argued that mandatory discounts under the 340B program force manufacturers to set higher list prices to achieve revenue targets,^5^ essentially shifting the value of the 340B discount onto other payers, this cost-shifting theory presupposes that manufacturers are underpricing their drugs relative to what the market will bear, inconsistent with a profit-maximization strategy.^30,31^ Instead, our analysis suggests that a large 340B market share for a product may encourage manufacturers to offer discounts through reduced list prices rather than through PBM rebates, counter to the idea that 340B discounts lead to higher prices. Proposals to reduce the size of the 340B program may therefore diminish the 340B program’s potential to restrain price increases or encourage list price decreases. Policymakers should carefully consider the potential effects the 340B program may have on manufacturer pricing decisions as part of any efforts to revise the 340B program.

### Limitations

This study has several limitations. First, there could be errors in the matching process to identify 340B-eligible prescribers, and not all prescriptions by 340B-eligible prescribers may be filled under the 340B program. However, the relative distribution of 340B-eligible prescriptions across therapeutic classes in our analysis is consistent with the theory that high 340B-share therapeutic classes may have different pricing incentives, and we do not have reason to believe that any errors in matching would disproportionately affect one therapeutic class more than another.

Second, the Medicare data set used only includes prescribers with 11 or more claims for a particular drug. This limit means that prescribers who infrequently prescribe a medication will be omitted from the database. These omitted prescribers may vary from those included in their location, potentially skewing the estimated 340B market share for individual drugs or therapeutic classes. For example, the Medicare Part D Drug Spending Dashboard for 2016 reports 141 708 claims for Harvoni,^32^ but our database only included 104 750 claims. We cannot estimate any disparity in claims for Epclusa or Zepatier, however, because the dashboard only includes drugs with claims in both 2015 and 2016. However, we do not have reason to believe that the omitted prescribers inconsistently vary in the distribution of their 340B participation by drug or therapeutic category, supporting the variation in 340B market share observed across therapeutic classes.

Third, Medicare beneficiaries may see different prescribers than non-Medicare patients, and our estimates of the share of 340B-eligible prescriptions for a therapeutic class or individual product may not reflect the 340B-eligible share in the broader market. However, the variation observed in the Medicare data likely reflects the relative distribution of 340B-eligible share across therapeutic class by all payers even if the actual share within a particular therapeutic class may differ.

These possible limitations in precisely identifying the 340B-eligible share of prescriptions may affect our calculations for weighted average per-treatment manufacturer revenue and estimates of total revenues for manufacturers and 340B health care organizations. However, the limitations do not affect the individual per-treatment revenues for manufacturers and 340B health care organizations before and after the price change, as these are fixed based on the 340B discount formula. Therefore, although our estimates of the total revenue could be affected by any misestimation of 340B share, the per-treatment revenue effect that suggests a market-based reason for manufacturer list price reductions is unaffected.

## Conclusions

In markets where an above-average share of prescriptions is eligible for 340B discounts, such as the HCV treatment market, manufacturers may prefer to reduce list prices rather than offer the same level of discount through PBM rebates. Such list price reductions increase manufacturer revenues while decreasing 340B health care organization revenues. Efforts to revise the 340B program should consider the possible effects on manufacturer drug pricing decisions.
