# Errors in Tables 3 and 4

**DOI:** 10.1001/jamanetworkopen.2019.11452

**Published:** 2019-08-23

In the Original Investigation titled “Estimated Changes in Manufacturer and Health Care Organization Revenue Following List Price Reductions for Hepatitis C Treatments,”^1^ published on July 5, 2019, there were 7 numeric errors in Table 3 in the “Mean” column and 8 numeric errors in Table 4 in the “Manufacturer Aggregate Revenue” column and 8 in the “340B Entity Aggregate Revenue” column. These table values did not match the values in the Results section. This article has been corrected.^1^
